# Clinical language fMRI with real-time monitoring in temporal lobe epilepsy: Online processing methods

**DOI:** 10.1016/j.yebeh.2012.05.019

**Published:** 2012-09

**Authors:** E.J. Williams, J. Stretton, M. Centeno, P. Bartlett, J. Burdett, M. Symms, J.S. Duncan, C. Micallef

**Affiliations:** a MRI Unit, Epilepsy Society, Buckinghamshire, SL9 0RJ, UK; b Department of Clinical and Experimental Epilepsy, Institute of Neurology, University College London, WC1N 3BG, UK

**Keywords:** Language fMRI, Language mapping, Epilepsy surgery, Real time, BrainWave, Online, Language lateralization

## Abstract

The increasing demand for clinical fMRI data has resulted in a need to translate research methods to clinical use. Referrals for language lateralization prior to epilepsy surgery are becoming more common, but time constraints make this unachievable in many busy neuroimaging departments. This study examines whether a single covert verbal fluency paradigm with real-time monitoring and online processing (BrainWave) could replace conventional offline processing (SPM) for the purpose of establishing expressive language dominance prior to epilepsy surgery. We analyzed language fMRI results of 30 patients (17 female; 24 right‐handed; median age: 30.5) with temporal lobe epilepsy. Concordance between visual assessment of SPM and BrainWave was 92.8%. Lateralization indices correlated closely with visual assessments of lateralization with a concordance of 85.7%. BrainWave provided a real-time, fast and accurate display of language lateralization easily applied in a clinical setting using only online image processing.

## 1 Introduction

Functional magnetic resonance imaging (fMRI) is widely used to map language activation and evaluate hemisphere dominance for language prior to epilepsy surgery. Numerous studies have shown fMRI to be a valid replacement for the intracarotid amytal test [1–3]. Functional magnetic resonance imaging has the advantage of being safe, noninvasive and repeatable. In addition, it can provide data on the intrahemispheric localization of language.

The increasing demand for clinical fMRI data has resulted in a need to translate research methods to clinical use. Routine referrals for language lateralization prior to epilepsy surgery are becoming more common, but the large amounts of data and the lengthy post-processing times used in research procedures make this unachievable in many busy neuroimaging departments. In the clinical environment, methods need to be quick, reliable, easy to implement and without special equipment.

The verbal fluency paradigm was selected for its ease of understanding for a wide range of patients with epilepsy with varying levels of cognitive abilities. It has been shown to reliably lateralize expressive language [4,5]. Following left anterior temporal lobe resection, up to 40% of patients will develop notable language deficits, particularly a decline in naming ability [6]. Verbal fluency tasks usually generate stronger and wider activations than verb‐generation tasks [7].

Most fMRI post processing requires data to be transferred offline. It is time‐consuming, requiring many hours of input by highly skilled operators and access to large amounts of disc space. Statistical parametric mapping (SPM), a widely used standard processing tool, is our routine method for language fMRI processing. In conjunction with this, we have applied BrainWave, a real-time and online fMRI‐processing package developed by the MRI scanner manufacturer. This can be performed and processed in 15 min, with no requirement for offline post processing.

As with most widely used fMRI, BrainWave uses the phenomena of blood-oxygen level dependent (BOLD) contrast [8]. It is only suitable for block design paradigms. BrainWave has the additional advantage of task‐performance monitoring with real‐time activation maps, so the need to repeat a scan due to poor task performance is known while the fMRI acquisition is taking place. Data quality is also monitored in real time using a traffic light system to alert the operator to poor data due to patient movement.

The main goal of this study was to compare two image processing systems, the current, widely used standard processing tool, SPM8 (Wellcome Centre for Neuroimaging techniques) and BrainWave (General Electric Healthcare 2003), to establish whether fMRI with online processing could replace conventional offline SPM processing for the purpose of establishing expressive language dominance prior to epilepsy surgery.

## 2 Materials and methods

### 2.1 Patients

Thirty consecutive patients (17 female) with temporal lobe epilepsy (TLE) who had all been referred for presurgical fMRI evaluation of language dominance were studied. The diagnosis of TLE and its lateralization was established by prolonged video/EEG monitoring and neuroimaging. All the patients completed a questionnaire to establish handedness [9]. Six patients were left‐handed, and 24 were right‐handed. The median age was 30.5 yrs (range: 18–59). Sixteen patients had a diagnosis of right TLE, 12 had left TLE and 2 had bilateral changes on EEG. Structural MRI showed hippocampal sclerosis (7), amygdala sclerosis (1), cavernoma (4), dysembryoplastic neuroepithelial tumor (DNET) (3), focal cortical dysplasia (5) and MRI negative (11) (Table 1).

All the patients gave informed written consent. This study was approved by the Research Ethics Committee of the National Hospital for Neurology and Neurosurgery and the UCL Institute of Neurology.

### 2.2 MR data acquisition

All scans were performed on a 3 T GE Signa Excite HD scanner (GE Medical Systems, Milwaukee, Wisconsin) at the Epilepsy Society MRI Unit. All data were acquired using an eight-channel array head coil for reception and the body coil for transmission. For the fMRI task, gradient-echo planar T2*‐weighted images were acquired providing blood oxygen level-dependent (BOLD) contrast. Each volume comprised 50 2.4/0.1‐mm oblique axial slices through the whole brain with a 24-cm field of view, 64 × 64 matrix and in-plane resolution of 3.75 × 3.75 mm. Echo time (TE) was 25 ms, and repetition time (TR) was 2.5 s.

### 2.3 Verbal fluency fMRI paradigm

A variant of a phonemic fluency task was employed [10]. The subjects viewed a single letter projected onto a screen at the end of the scanner couch via a prismatic mirror as they lay in the scanner. The subjects were instructed to covertly generate words in response to the visually presented letters (A, S, W, E and D). Each active condition was presented in blocks lasting for 30 s with ten presentations of a given letter per block and an interstimulus interval (ISI) of 3 s. The active condition was alternated with a 30 s control condition. Five blocks of each condition were performed. In total, the acquisition lasted for 5 min and 10 s.

### 2.4 Scanning and real-time monitoring

Before entering the scan room, the patient was consented and given a verbal explanation and a visual demonstration of the language task. A brief final reminder of the task was given immediately before the scan began.

Patient compliance was monitored in real time during the verbal fluency task using the BrainWave software. The 30‐second block design paradigm was alternated between the verbal fluency task and rest, beginning with rest. After the first 30‐second task, real-time activation images were viewed on the scanner console by selecting a *t*-test from the statistical analysis options. Activation maps created in real time by BrainWave were viewed directly on the raw echo-planar imaging (EPI) data or on a high‐resolution EPI scan acquired immediately before the functional scan. Activations built up over time and were saved to the scanner disc in DICOM format during or at the end of the acquisition.

The real-time activation plots gave assurance that meaningful data were being collected. Poor task performance was immediately evident, and the task could be repeated, if necessary, after another brief explanation. A data quality algorithm in BrainWave monitored signal‐to‐noise ratio (SNR), ghosting and patient movement and presented the results in real time with a green/yellow/red traffic light display on the real-time viewing console. If preset limits are exceeded, the light turns to red and the operator can stop the scan and coach the patient to assure high-quality EPI data (General Electric Healthcare 2003).

A 3D T1 Volume (FSPGR; 1.1 mm/256 × 256/1 NEX/24 FOV) was acquired within the same examination. BrainWave post processing was performed online at the scanner console and took 3 min. Echo‐planar imaging images were co‐registered with the T1 volume displayed in all three orthogonal planes and saved to the scanner disc. The default Z score and P value used for thresholding were Z > 4.56 (P = < 0.05). Thresholds could be altered by a simple reprocessing step. A preliminary visual assessment of language laterality could be made before the patient had left the scan room.

## 3 Data analysis

### 3.1 SPM analysis

Imaging data were analyzed using statistical parametric mapping (SPM8). The imaging time series was realigned and smoothed with a Gaussian kernel of 8 mm full-width‐half‐maximum. For each subject, trial-specific responses were modeled by convolving a delta function that indicated each active block onset with the canonical hemodynamic response function (HRF) to create regressors of interest. Each subject's movement parameters were included as confounds, and parameter estimates pertaining to the height of the HRF were calculated for each voxel. One contrast image for the main effect of fluency was created for each subject. The rest condition was used as baseline. We report all activations at a threshold of P < 0.05.

### 3.2 BrainWave analysis

BrainWave real-time online image processing applied motion correction by registering all of the scans in the analysis data set to the same reference scan. Functional magnetic resonance imaging images were aligned using Woods AIR method [11] to minimize movement artifact. A motion correction plot indicated the magnitude and direction of rotations and translations detected and corrected during realignment. The fMRI image volumes were smoothed with a Gaussian spatial filter of full-width-half-maximum (FWHM) of 8.0 × 8.00 × 8.0 mm. Scans were then analyzed on a voxel‐wise basis using multiple regression (general linear model) generating a *t*-test map. The method of Worsley and Friston [12] was used to estimate the effective number of degrees of freedom, to account for temporal autocorrelations due to the smoothness of the hemodynamic response. Using the estimate for the number of degrees of freedom, the *t*-test was converted into an activation Z map. The activation map was then co-registered to the segmented structural T1 volume series.

### 3.3 Image display

The activation maps, co-registered with the segmented structural T1 volume were created in 3 orthogonal planes and saved as an image stack within the patient directory along with the other structural scans. An overview of the entire brain was also archived with areas of activation displayed on the 3D‐segmented volume. The images were transferred to a satellite work station for viewing and reporting.

### 3.4 Language maps rating

Statistical parametric mapping and BrainWave images were assessed independently by two raters, a neuroradiologist specializing in fMRI (CM) and a neurologist specializing in fMRI (MC). The images were anonymized, and the raters were blinded to any clinical information. Areas of activation were divided into middle and inferior frontal gyri, and superior and middle temporal gyri. Visual assessment of significant activations in each of these areas was noted followed by an overall visual assessment for left, right or bilateral language dominance. In addition, left‐ and right‐sided activations in the cerebellum were noted; cerebellar activation in the contralateral side to that of language dominance has been noted [13,14]. A quantitative assessment of lateralization indices (LI) of activation in the middle and inferior frontal gyri (MFG/IFG) was performed for comparison with the visual radiological assessment. We calculated the LI for the MFG/IFG using the bootstrap method of the SPM toolbox [15] for the contrast “verbal fluency” for each subject (− 1 for left hemisphere activation and + 1 for right hemisphere activation).

## 4 Results

Two of the 30 patients were excluded because of poor data quality. There was good concordance between SPM and BrainWave for the remaining 28 patients (Table 2).

Rater one assessed laterality of verbal fluency the same for SPM and BrainWave activation maps in 26 subjects and with some variation in 2 patients (concordance: 92.8%). A 41‐y/o subject with RHS and right temporal dysplasia was reported as left dominant with some right activation on BrainWave but was reported as bilateral on SPM (lateralization index: − 0.51). A 46‐y/o female with RHS was reported as right dominant on BrainWave and bilateral but with slightly more activation on the right with SPM analysis (lateralization index: 0.25).

Rater two reported 25 of the 28 subjects the same on SPM and BrainWave (concordance: 89.2%). A 32‐y/o female with RHS was reported as left dominant but with some right activation on BrainWave and bilateral on SPM (LI: − 0.0058). The same 41‐y/o subject with RHS and right temporal dysplasia (rater 1, above) was reported as bilateral on SPM and left dominant on BrainWave with some right activation noted. A 59‐y/o female with RHS was judged to be bilateral with significant right activation on BrainWave and right lateralized on SPM (LI: − 0.038).

In the cases in which the 2 raters disagreed individually, a consensus was reached, and the concordance was 92.8% (Tables 1 and 2).

### 4.1 Comparison of visual reading with lateralization index of activation in the middle and inferior frontal gyri

**Fig. 1:**
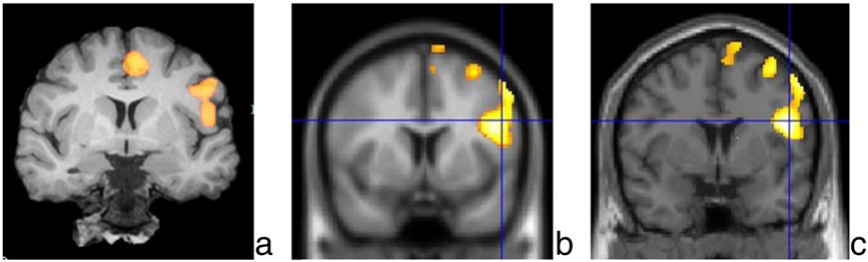
(a) BrainWave coronal activation maps showing left‐sided activation in the inferior and middle frontal gyri of a left‐handed 27‐y/o female with right TLE and right amygdala sclerosis. (b) Statistical parametric mapping activations mapped onto EPI data set and (c) coronal T1‐weighted image (LI: − 0.93). The left side of the brain is displayed on the right side of each image.

**Fig. 2:**
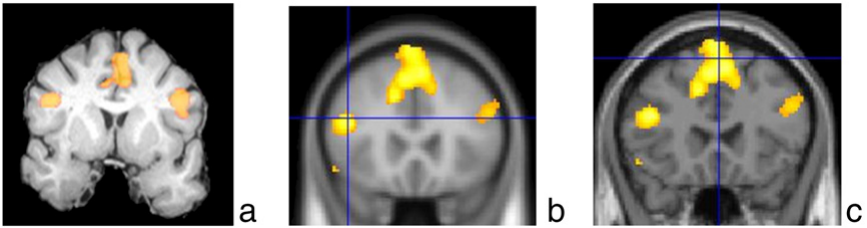
(a) BrainWave coronal activation maps showing bilateral activation in the inferior frontal gyrus of a 46‐y/o left‐handed female with right TLE and right HS. (b) Statistical parametric mapping activations mapped onto EPI data set and (c) coronal T1‐weighted image (LI: 0.25). The left side of the brain is displayed on the right side of each image.

Left language dominance was defined by an LI of ≤− 0.4 on the verbal fluency task. Right language dominance was defined by an LI of ≤+0.4. The range of LI was between − 0.99 and + 0.72. Lateralization indices correlated closely with visual assessments of lateralization (Table 1, Figs. 1 and 2). There were 2 cases which were not concordant between BrainWave and SPM, and these were compared with SPM‐derived LIs. Patient 22 had an LI of − 0.51 and was visually assessed to be left dominant with BrainWave and bilateral with SPM. Patient 27 had an LI of 0.25 and was assessed to be bilateral on SPM but right dominant on BrainWave.

Patients regarded as left dominant using SPM had an LI of − 0.22 to − 0.99. Patients regarded as left dominant using BrainWave had an LI of − 0.22 to − 0.99. The patient regarded as right dominant using SPM had an LI of 0.70. The patients regarded as right dominant using BrainWave had an LI of 0.25 to 0.70. Patients regarded as bilateral using SPM had an LI of − 0.51 to 0.25. Patients regarded as bilateral using BrainWave had an LI of − 0.38 to − 0.0058 (Table 1). This demonstrates that visual reading of the SPM and BrainWave images gives a good correlation with the LI measures.

## 5 Discussion

There was good concordance between the two blinded raters for BrainWave and SPM. Inconsistencies were due to some degree of bilateral asymmetric dominance. A consensus was reached between the raters, and the concordance between the two methods was 92.8%. The two remaining cases with inconsistencies between SPM and BrainWave were in those patients with bilateral asymmetric activations.

Lateralization indices correlated closely with visual assessments of lateralization; the concordance was 85.7%. Visual assessment of BrainWave was just as accurate as the visual assessment of SPM when compared with LI. The discrepancies were in cases with degrees of bilateral activation.

Two patient data sets were excluded due to non-diagnostic results on SPM. BrainWave analysis coped well with both movement artifact in the first case and poor activation in the second case. Results on BrainWave for both cases were considered diagnostic. In two other cases, there were discrepancies between visual assessment and LIs. Visual assessment of SPM and BrainWave concurred as left dominant in both cases; however, the LI reading suggested bilateral representation. In one case, there was artifact which was disregarded by the visual assessment but which contributed to the bilaterality of the LI. In the other, the data quality was suboptimal, and a repeat acquisition may have given a clearer result.

### 5.1 Comparison with previous work

Although scanner‐based online fMRI‐processing packages with real-time monitoring have been available for some time, little has been written about their validity as an alternative to offline processing methods. In 1995, Cox et al. [16] recognized that the capacity for real-time viewing of fMRI data was desirable for several reasons: 1) for data quality monitoring and motion detection, the need for repeat tasks would become immediately obvious; 2) instant access to initial results would make it possible to develop new paradigms more quickly; 3) interactive paradigms would become possible. Weiskopf et al. [17] recognized the value of immediate quality assurance and functional localizers to guide the main experiment, which real-time fMRI analysis provides.

Fernandez et al. [18] studied 12 patients and 12 control subjects using a semantic decision task to evaluate language lateralization in epilepsy patients. They concluded that real-time analysis was a reliable method for assessing language dominance. Several limitations in real-time processing were highlighted; having to set a predefined statistical threshold a priori and the inability to register activation maps with structural anatomical images. Both limitations have been overcome with the current real-time package used in this study; statistical thresholds can be altered by redefining a Z-score value during image processing and viewing. Activation maps can be overlaid in real time with a high‐resolution EPI scan acquired in the same plane with the same slice thickness immediately before the task. In addition, overlay with a structural high‐resolution T1 volume is performed immediately after the functional acquisition.

Schwindack et al. [19] compared real-time activation maps with standard offline SPM results in 11 patients with brain tumors. For the real-time analysis, Schwindack used an adapted version of AFNI software (National Institute of Mental Health, Bethesda, Maryland, USA) customized to their GE scanner. They found that motor finger‐tapping tasks provided the most consistent activation between the two methods, but they had less success with real-time language paradigms and, thus, recognized the need for further studies.

### 5.2 Clinical interpretation of results

The presurgical determination of language dominance is required to predict and minimize the risk of language deficits after epilepsy surgery. Left TLE is associated with a higher incidence for atypical language dominance compared to healthy controls and right TLE [20]. Atypical dominance is most likely to occur with onset of epilepsy in childhood [21].

Following left anterior temporal lobe resection, up to 40% of patients will develop notable language deficits, particularly a decline in naming ability [6]. Preoperative language fMRI has been shown to predict marked language decline with increasing activation in the left hemisphere, particularly in the temporal lobe, being associated with increasing risk of postoperative impairment [22]. Left-sided TLE is associated with an increased probability of expressive language activation in the right frontal lobe [20].

As with all fMRI, caution is required in the clinical interpretation of results, whether from BrainWave or SPM analysis. Hemispheric language dominance is not dichotomous but follows a continuum, so individual cases are not always clear-cut. It is helpful to use a laterality index to express language laterality as a continuous variable rather than left, right or bilateral. It has been noted that language lateralization with fMRI might be less reliable in the presence of a structural lesion than without [23]. Functional magnetic resonance imaging results cannot be used to determine the extent of neocortex that is needed to subserve language, the area shown being consequent to the thresholds used to display the results. Direct cortical stimulation is required as an additional preoperative assessment to precisely define critical language cortex if surgery is planned close to this area.

### 5.3 Future studies

We have also had success using online BrainWave image processing for other language paradigms. Verb‐generation tasks can be modified to a simple block design suitable for real-time scanning; initial results show good concordance with SPM processing, but more data are needed for future comparison. Motor tasks are ideally suited to real-time scanning; immediate activation maps co‐registered with the T1 volume provide useful and instant information on structural proximity of the lesion to the motor cortex. Future validation studies are needed in these areas. BrainWave is just one of several commercially available online fMRI‐processing packages suitable for setting up a clinical fMRI language service. Brainlab, AFNI and BrainVoyager are alternative packages produced by other manufacturers.

## 6 Conclusions

Online image processing using BrainWave provided a fast and accurate display of expressive language lateralization in presurgical epilepsy patients. It can easily be applied in a clinical setting, without the need for intensive offline data processing. BrainWave showed good concordance with the current standard offline method for fMRI analysis, SPM. Real-time activation plots gave assurance that meaningful data were being collected. Cases of poor task performance were immediately evident on BrainWave activation maps allowing for task repetition during the same examination. BrainWave reliably identified typical left language dominance and highlighted atypical cases that may require offline post processing for full clinical evaluation.

## Figures and Tables

**Table 1:**
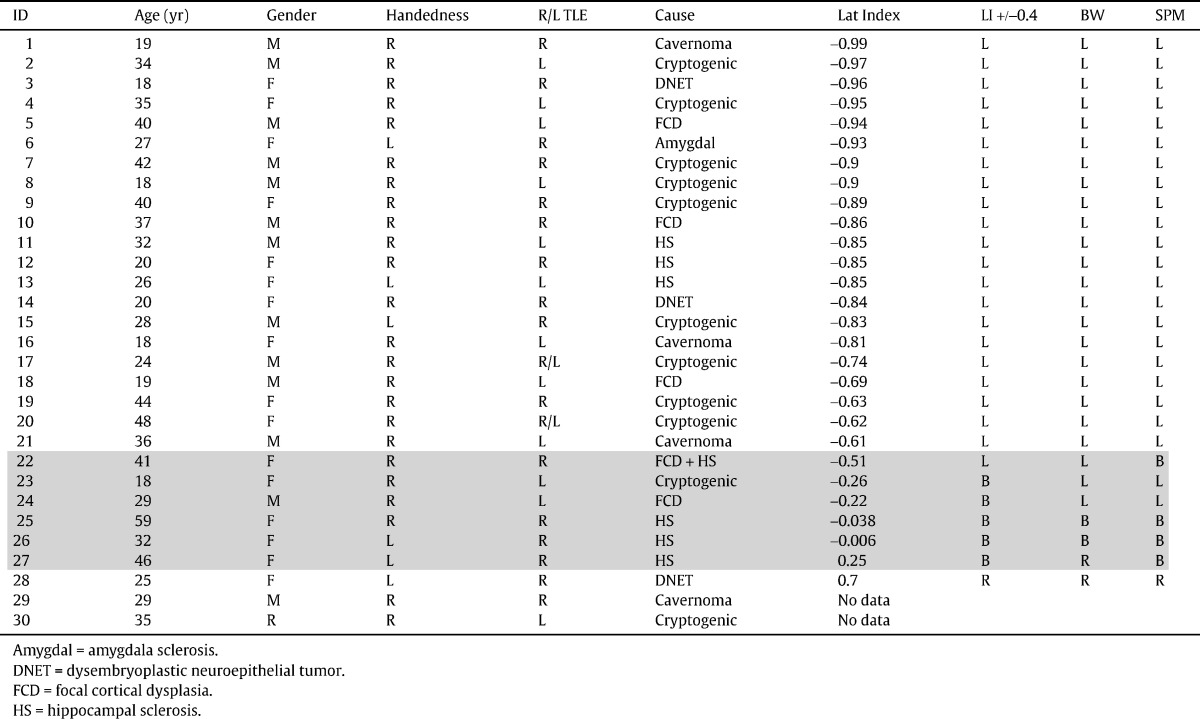
Clinical patient data and lateralization indices for the inferior and middle frontal gyri compared with the visual assessment of lateralization using SPM and BW analyses. Shaded areas show bilateral representation in one or more assessment methods.

**Table 2:** Visual assessment: results of SPM and BrainWave for language lateralization.

		Left	Right	Bilateral
Rater 1	SPM	23	1	4
BrainWave	24	2	2
Rater 2	SPM	23	3	2
BrainWave	25	2	1
Consensus	SPM	23	1	4
BrainWave	24	2	2
